# Parsley Health: Feasibility and acceptability of a large-scale holistic telehealth program for chronic disease care

**DOI:** 10.3389/fdgth.2023.1008574

**Published:** 2023-03-15

**Authors:** Hants Williams, Sarah Steinberg, Ryan Vingum, Kendall Leon, Elena Céspedes, Robin Berzin, Heather Hagg

**Affiliations:** ^1^ School of Health Professions, Stony Brook University, Stony Brook, NY, United States; ^2^ Parsley Health, New York, NY, United States; ^3^ Untold Content, Cincinnati, OH, United States

**Keywords:** chronic conditions, holistic medicine, telehealth, patient engagement, healthcare system

## Abstract

**Background:**

A holistic, personalized approach to medicine can be used to prevent and manage a variety of chronic diseases. However, effectively managing chronic diseases can be difficult due to barriers related to insufficient provider time, staffing, and lack of patient engagement. To address these challenges telehealth strategies are being increasingly adopted, yet few studies have explored how to evaluate the feasibility and implementation success of large-scale holistic telehealth models for chronic disease care. The aim of this study is to assess the feasibility and acceptability of a large-scale holistic telehealth program for the management of chronic diseases. Our study findings can inform the future development and assessment of chronic disease programs delivered through telehealth strategies.

**Methods:**

Data was collected from participants enrolled in a Parsley Health membership from June 1, 2021 to June 1, 2022, a subscription-based holistic medicine practice designed to help people prevent or manage chronic diseases. Implementation outcome frameworks were used to understand engagement with services, participant satisfaction, and preliminary effectiveness of the program *via* a patient-reported symptom severity tool.

**Results:**

Data from 10,205 participants with a range of chronic diseases were included in our analysis. Participants averaged 4.8 visits with their clinical team and reported high levels of satisfaction with their care (average NPS score of 81.35%). Preliminary evidence also showed substantial reduction in patient reported symptom severity.

**Conclusion:**

Our findings suggest the Parsley Health program is a feasible and acceptable large-scale holistic telehealth program for chronic disease care. Successful implementation was due, in part, to services that promoted participant engagement along with tools and interfaces that were helpful and easy to use. These findings can be used to develop future holistic-focused telehealth programs for the management and prevention of chronic diseases.

## Introduction

Chronic diseases, or conditions lasting more than a year that require regular medical attention, have become an epidemic in the United States. With over 60% of the U.S. population living with at least one chronic disease, care for these conditions accounts for about $3.15 trillion of the $3.5 trillion in annual health care spending in the U.S. (1). Epidemiological data indicates that common chronic diseases, such as diabetes, heart disease, and asthma, are likely underdiagnosed up to 90% of the time in the developed world (2). With the 65-and-over population growing, coupled with increased risk factors, chronic disease prevalence has continued to grow every year over the past decade (3). The rise in chronic disease prevalence and its correlative financial and societal burden has exposed the inability to effectively monitor, manage, and prevent these conditions within the confines of the traditional healthcare system (4, 5). Research shows that people with chronic conditions require more care for their conditions than our current system allows (6), leading to more reactive than proactive care (7), more prescription drugs (8), higher costs (9), and overall lower health outcomes (10).

Some models have emerged as solutions to the limits of the traditional health care system. These care models include holistic care programs that focus on different dimensions of a person's health to inform preventive care (11–13) and programs informed by the Chronic Care Model (CCM) (14–16), which focuses on restructuring healthcare systems to emphasize proactivity and improve patient engagement in their care. However, many time and resource constraints in traditional systems remain obstacles to effectively implementing care models and clinical preventive services proven to help reduce chronic disease rates and improve outcomes (17).

Telehealth, or the use of technology to deliver resources and health care services, has proven to be an effective way to deliver chronic care programs in a traditional health care setting (18). Telehealth strategies can overcome many time and resource constraints by supporting more collaborative disease management, increasing patient engagement, improving patient self-management of conditions, decreasing medication use and hospital visits, and lowering healthcare spending (19–21). However, the use of telehealth for chronic disease management is still an emerging trend, despite explosive growth since the start of the COVID-19 pandemic and a growing desire among patients/consumers to be more active in their care, and for more care options. Ultimately questions remain about the best ways to deliver chronic care interventions *via* telehealth and overcome the limitations of existing healthcare systems.

Parsley Health was founded in 2016 in response to the demand for greater access to holistic care and the limits of existing care models to deliver preventive care for chronic conditions. Parsley Health's care program is a team based, membership model designed as an innovative way for patients and providers to leverage telehealth tools to navigate the prevention and management of complex chronic diseases through a holistic medicine lens, with an emphasis on patient engagement and ownership in their care. Patients receive care in-person or through virtual visits, and all patients have access to various digital tools and interfaces to support them on their healthcare journey. The program incorporates two medical approaches to care: standard care (e.g., proactively managing the need for prescription medications and specialist care) and functional and holistic medicine (e.g., prioritizing nutrition, wellness, and lifestyle medicine). This study explores feasibility and implementation-related metrics from the patient perspective to understand the impact of the Parsley Health program design and delivery.

## Materials and methods

### Participants and recruitment

Study participants were active members of Parsley Health between June 1, 2021–June 1, 2022. Selection criteria included those who were 18 years or older, in their first year of their membership, or had renewed their membership during the study time period.

**Figure 1:**
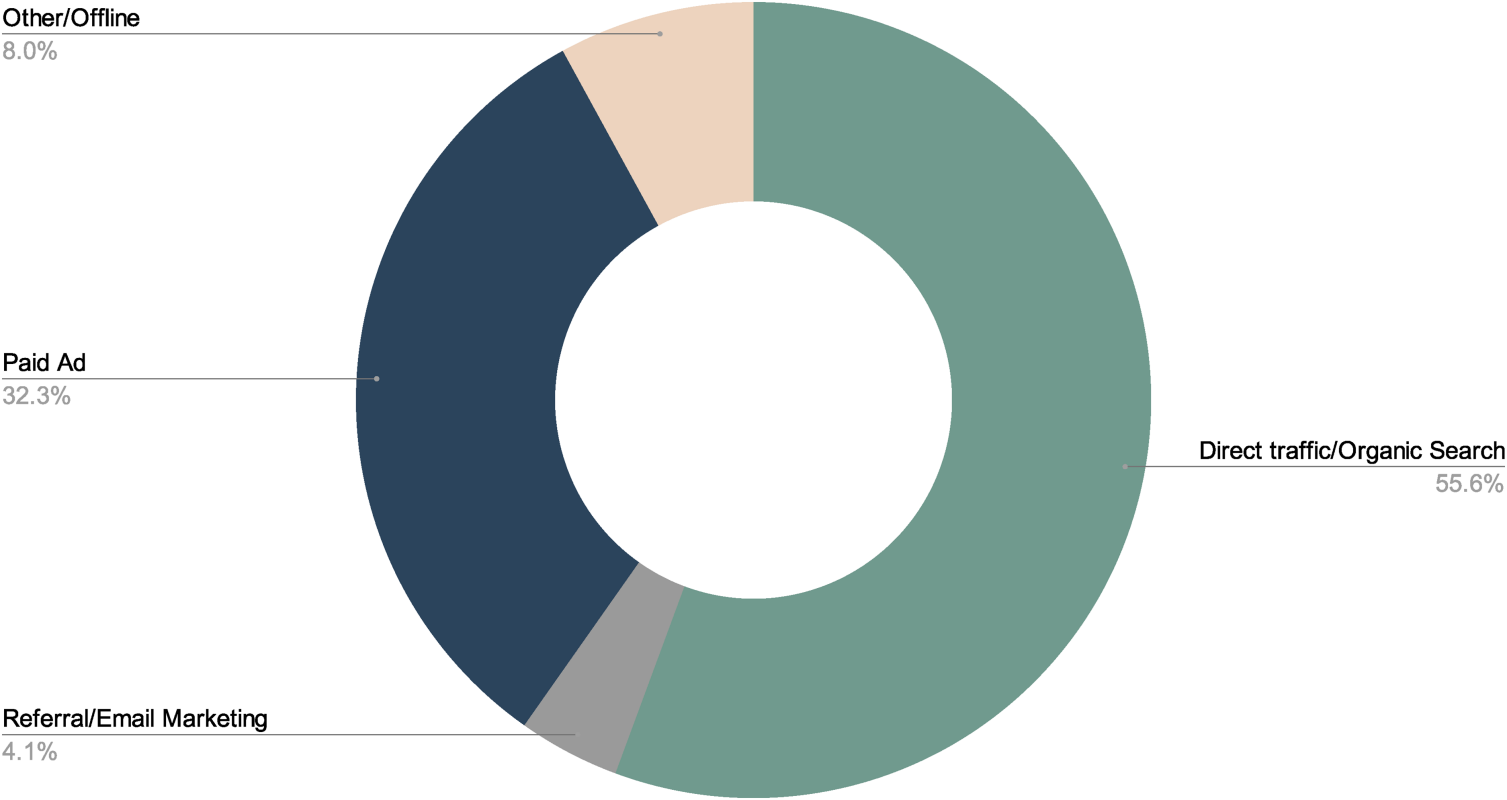
How patients find parsley health.

Parsley Health used traditional and nontraditional marketing methods for outreach to attract new members and potential participants. While some patients were referred to Parsley through word-of-mouth (22), the majority found their way to Parsley Health digitally. Deidentified data in Figure 1 shows that that over half of participants found Parsley Health through an organic search (e.g., typing a search query into a search engine). About 36.4% of participants found Parsley Health through some kind of active advertising strategy, such as ads or email marketing. Digital marketing strategies are essential in a world where smartphones are nearly ubiquitous, yet the healthcare field is behind in its use of digital marketing (23), and can prove useful for reaching patients looking for new chronic care options. Individuals interested in joining Parsley Health used an online portal to sign-up. At this initial point of contact, individuals encountered a digital space designed to set them at ease (24).If potential patients contacted Parsley Health to learn more before enrolling, they interacted with an employee on the “member experience” team who answered questions about membership logistics and costs. The team member could also help patients choose a provider with appropriate clinical expertise, and one who may be a good personality fit. If concerns with any aspect of their care arose during their membership, patients worked directly with an employee on this membership experience team. This provided another level of support to patients and allowed care teams to focus their time on clinical care.

### Program design

The program described in this paper was a 12-month subscription model service. The membership structure allowed for more visits, robust healthcare monitoring, and multiple levels of accountability to support behavior changes. The main membership program, called Complete Care, included up to ten visits in a year - five clinician visits and five health coach visits.

Clinicians were medical doctors (MDs), Doctors of Osteopathy (DOs), Physician Assistants (PAs), or Nurse Practitioners (NPs) while health coaches held degrees or certifications in nutrition, coaching, and dietetics. All clinical care team members underwent a rigorous 12-week training “fellowship” prior to starting in the care program. The fellowship focused on holistic approaches to medicine with a focus on digestive diseases, autoimmune disease, cardiometabolic disease, and endocrine disorders. After joining the program, patients were assigned a care manager. Care managers were responsible for scheduling assistance, answering logistic inquiries, and handling email triage.

Clinician meetings were designed to allow for an in-depth clinical assessment. A clinician could examine the root cause of a patient's symptoms, formulate a treatment plan, and tailor that plan depending on the participant's response to different interventions. Health coach meetings were designed to support the patient's lifestyle change goals (nutrition, sleep, stress management, and exercise) to support their medical goals.

After the first year, patients could select from several renewal plans, which allow more flexibility. Patients chose from programs with 3–5 clinician visits and 3–5 health coach visits, depending on their need for follow-up frequency.

Prior to the COVID-19 pandemic, initial visits were in person and patients had the option to choose a virtual or in person follow up. Since the start of the COVID-19 pandemic, all visits—whether with a clinician or a health coach—took place online over Zoom. The Parsley Health program emphasized design of the clinical and digital spaces to ensure all patients receive a similar experience regardless of the modality. Similar to the physical space of the Parsley Health clinics, the digital interface was designed to create a welcoming and calming environment to immediately activate patient satisfaction with their first encounter with a healthcare practice (25). As the pandemic evolved, patients had the option to return to in-person visits or continue telehealth services. For patients utilizing telehealth services in this study, care managers helped address any technical issues they may experience. While clinical visits have shifted between completely virtual and a hybrid option of virtual or in person, all other clinical touch points—onboarding, intake forms, clinician messaging, and portal engagement—have been digital from the beginning and were specifically designed for a virtual experience. All patients were given on-boarding documentation and intake forms to prepare for their first visit. These forms (which were all completed digitally) asked about their medical history, lifestyle habits, nutritional habits, and health goals. Among the intake forms participants completed was the Parsley Symptom Index (PSI), a digital, clinically-validated (26), proprietary electronic patient reported outcome measure (ePROM) used to evaluate a member's symptoms in several different areas (e.g., cardiac, digestive, respiratory). The PSI was designed specifically to support member and clinician engagement and collaborative care management in a telehealth environment. Patients were asked to complete it regularly before every clinician and health coach visit.

The PSI included a unique graph feature that allowed patients and providers to track responses over time and by different body systems. With regular use, the PSI created opportunities to share data between patients and clinicians and improve communication. Patients could access their PSI graphs digitally at any time and note any significant changes they saw in their own health to help identify triggers and mediators of symptoms. The graph could also help patients make connections between their symptoms and specific behaviors or treatments, promoting participant accountability during the care process.

**Figure 2:**
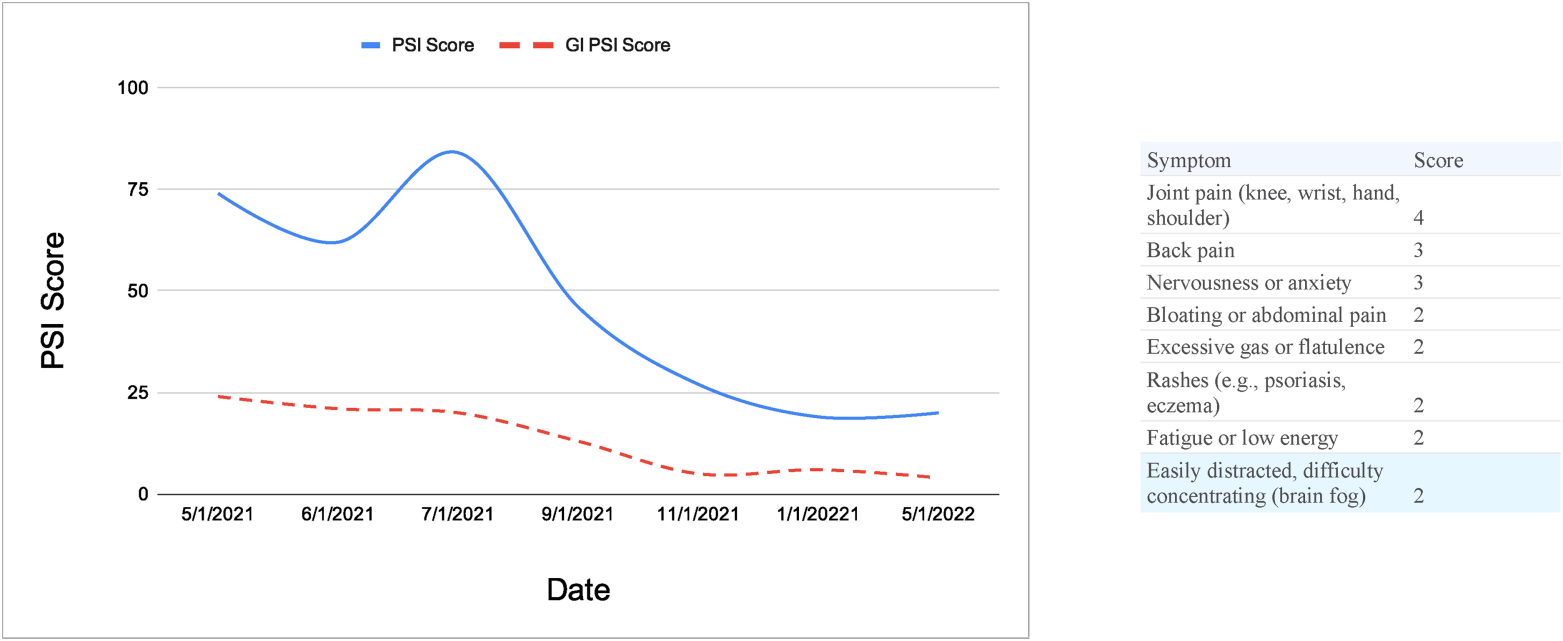
PSI mockup view: symptom changes tracked over time.

Figure 2 is a mockup of how a patients PSI scores appear on a member facing visualization. This mockup shows what symptom changes look like for a typical member after successful treatment for small intestinal bacterial overgrowth (SIBO), a common ailment treated at Parsley Health. The higher the PSI score, the more severe the symptoms a member reported experiencing. These graphs can also be filtered by the body system to show a patient their progress in a specific area.

PSI results were integrated into a patient's electronic health record (EHR), giving clinicians and health coaches easy access to a member's self-reported wellbeing. As prior research suggests, this integrative capability allows members and clinicians to review ePROM results before a clinical visit as well as during the visit—with the patient—resulting in more targeted, collaborative conversation (27).

In the initial visit, the clinician and patient discussed intake forms, including the PSI. The clinician also conducted detailed medical history, physical exam, and discussed the immediate and long-term plan. This meeting differs from typical medicine. Notably, intake forms and medical history information are used to identify triggers that led to chronic conditions. In contrast, the typical approaches to chronic disease management treat each individual chronic condition symptomatically, but not necessarily with the goal of reversing or diminishing the cumulative disease burden. About two to four weeks following the initial visit with a clinician, patients had their first meeting with a health coach. During this visit, patients worked with their coaches to begin setting health goals and strategies to meet these goals.

After these initial visits, patients continued to see their clinician and health coach on a regular basis. Follow up was recommended every 10–12 weeks, but varied depending on individual needs. Prior to all visits, patients were prompted to complete the PSI electronically so that patients and clinicians could review the results on PSI graphs before the visit. During follow-up visits, clinicians reviewed any lab or test results, discussed a member's health concerns, and worked to set treatment goals. These frequent visits and regular health data collection allowed members to build close relationships with their care team. Relationship continuity and trust (28) in a healthcare setting leads to better health outcomes, especially for people with chronic conditions (29, 30). Health coaches keep patients connected and engaged with their care (31) and play a vital role in helping patients make positive behavioral changes, improving satisfaction with care (32–34).

Patients in the early stages of a chronic disease had access to a range of preventive care services, which helped avoid visits to a specialist outside their established care network. The model described in this paper differs from a typical approach to medicine in many ways. After the patients complete extensive onboarding forms collecting detailed information about their past medical history from birth to current day, they have their first hour long intake appointment where specific attention is paid to disease triggers that preceded the onset of chronic disease. Part of preventive care in this program involves providing patients with an option for in-depth lab testing, including advanced cardiac marker testing, as well as specialty (out of pocket) testing, such as stool microbiome, SIBO, urine hormone, and heavy metals/toxins testing. By offering robust testing options, clinicians and patients could explore the root cause of a condition, track treatment progress, and make informed, data-driven care decisions. Follow up visits include extensive discussion and explanation of conventional lab results as well as specialty testing. The goal was to help patients with pre-diabetes, subclinical hypothyroidism, hyperlipidemia, or early signs of other chronic diseases improve their symptoms or normalize their lab results through diet and lifestyle changes without needing to visit an external specialist. After their first two visits, the assessment and then the lab review, patients have a detailed plan designed specifically for their symptoms, with a focus on disease triggers and the level of lifestyle change that's right for them. These plans are supported by virtual coaching visits in between clinician visits. Generally speaking, patients are coached on lifestyle changes addressing diet, physical activity, sleep, and stress management before any prescriptions are made. Diet and supplement recommendations, backed by peer reviewed evidence, are often recommended before common prescription medications are prescribed.

To leverage care team support and health data, patients utilized MyParsley, an online portal where they could review lab test results, read visit notes, and access educational materials. The ability to share data between patients and multiple providers can improve the quality of care a patient receives (7). Patients also had unlimited messaging access through the portal, allowing them to get any treatment plan questions answered quickly or to update their team on any health changes. Technologies such as personal messaging options can improve regular contact with a provider, decrease patient confusion, improve adherence to treatment plans, and improve overall clinical outcomes for people with chronic conditions (35).

### Data collection and outcome measures

Studies examining the implementation of care or treatment programs have used a wide range of metrics to measure success, including clinical outcomes measures. We draw on implementation, service, and client-based outcomes (36) for measuring programmatic success and challenges, (as opposed to disease-specific treatment outcomes). Implementation outcomes, in particular, “serve as necessary preconditions for attaining subsequent desired changes in clinical or service outcomes” (36, p. 65–66). In other words, effective implementation of a program or treatment is key to its success. Implementation outcomes vs. treatment outcomes may also help better translate chronic disease care models and strategies from theory or laboratory settings into actual practice, allowing research results to be replicable by helping understand the processes for implementing certain programs.

Five outcome measures were selected and correlated to a specific element of the care program:

• Efficiency (time to first visit)
• Feasibility (visit utilization and PSI completion rates)
• Acceptability (engagement with MyParsley portal)
• Satisfaction (participant satisfaction with the program)
• Effectiveness (participant PSI score improvement)

*Efficiency**:*** Time to first visit is calculated by subtracting the first medical encounter date to the patients' activation date. Time to first encounter for a provider visit in the United States averages 19 days (approximately 2.7 weeks), with specialists ranging from 13.1 days (orthopedics) to 44.8 days (rheumatologists) (37). A time to first visit that is below the national average is interpreted as above average.

*Feasibility**:*** Visit utilization is calculated by taking the sum of medical encounters and health coach encounters for the study period One previous study calculated the average number of medical encounters (for primary care) in the U.S. to be 1.6 (38), which is substantially lower compared to other countries such as Germany (7.0) and the United Kingdom (5.4) (39). A visit utilization that is above the U.S. national average is interpreted as above average (i.e., good). PSI completion rates are calculated by the total number of completed PSIs by the total number of medical encounters. Previously reported completion rates of the PSI were 93.72% (26). A completion rate above 93% is interpreted as above average (i.e., good) for the Parsley Health population.

*Acceptability**:*** The MyParsley patient portal is a secure web application that allows a patient to access their health information, and message care team staff that include clinicians, health coaches, care managers, and member experience staff. Engagement with the MyParsley patient portal is measured by the number of average individual patient logins per month, and by the average number of messages sent between the patient and care provider staff per month (40). An active patient portal user is defined here as 8 or more monthly logins per month (41), or 3 or more messages per month (42–44).

*Satisfaction:* The Net Promote Score (NPS) is a simple survey question used to identify loyal customers (or patients) that is scaled from 0 to 10 (45). Healthcare companies have begun to incorporate the NPS tool into their clinics and hospitals to assess patient satisfaction with the provided services (46). The NPS is a single question: How likely is it that you would recommend our company to a friend or colleague? The item answer ranges from 0 (“not at all likely”) to 10 (“extremely likely”). Individuals that report a 9 or 10 are “promoters” that will give positive word-of-mouth advertising; persons that report 7 or 8 are considered indifferent (“passives”), while answering 0–6 are “detractors” that are likely to talk poorly about the services provided. The NPS score is then calculated as the percentage of promoters minus the percentage of detractors.

*Effectiveness***:** Effectiveness of the program was measured by changes in the Parsley Symptom Index (PSI), a recently developed symptom assessment for adults with chronic disease in telehealth settings (26). Items are grouped into 9 systems, with each containing 4 to 7 items per group that are ranked on a scale from 0 (asymptomatic) to 10 (extremely symptomatic). A total score is calculated with the following 4 cutoff ranges: 0–24, 25–43, 44–71, and greater than 71. The respective terminology for these ranges are “well” (0–24), “symptomatic” (25–42), “very symptomatic” (44–71), and “sick” (71+). For detecting changes within the PSI, a generalized linear mixed effects model (PROC Mixed Procedure; SAS version 9.4) with random subject effects to account for the correlation among repeated observations (47) was used to examine PSI score changes between baseline and subsequent follow ups, and to determine the predicted least-square (LS) mean values. Two strengths of the PROC MIXED procedure are that it considers an unequal number of measurements per participant for a given time period, and accounts for time intervals that are not constant between responses. A sub-sample of participants who had completed a baseline PSI response on or after the start of the study period (June 1st 2021) were included for the GLM. Non-renewal participants were excluded from this analysis because their baseline PSI scores were collected before the start of the study period. Participants were then placed into one of four group's based on their baseline PSI score: “well”, “symptomatic”, “very symptomatic”, or “sick” (article citation). PSI group (well, symptomatic, very symptomatic, or sick), time point (baseline, follow-up 1, follow-up 2, and follow-up 3), and the interaction between PSI group and time were included as fixed effects, and participants as a random effect (48).

Efficiency, feasibility, acceptability, and patient satisfaction help understand whether participants engaged with services provided by the program and whether these services were easy to use and access, key indicators of implementation success (49). If the program was not effectively implemented, we would expect that patients would find the program and interfaces challenging to use and there to be little to no engagement with services. While effectiveness, provides some insight into whether successful implementation of the care program led towards “desired clinical outcomes and changes,” as evidenced by symptom severity changes. More research is needed to study treatment-based outcomes of the program, however.

## Results

10,205 Parsley Health patients were included in this study (Table 1). Patients were predominantly female (84.2%) and white (55.3%). The most common condition groups (Table 2) were digestive issues (54.7%), mental health issues, such as anxiety, and depression (44.1%), cardiometabolic disease (39.4%), dermatologic conditions (38.3%), hormonal and/or fertility issues (36.7%), autoimmune diseases (19.9%) and thyroid disease (18.7%). Frequency counts by the International Classification of Diseases (ICD) are described in Table 3.

**Table 1:** Demographics.

	Level	Overall
*N*		10,205
Biological Sex at birth (%)	Female	8,590 (84.2)
	Male	1,467 (14.4)
	Other	148 (1.5)
Gender Identity (%)	Woman	6,331 (62.0)
	Man	996 (9.8)
	Non-Binary	66 (0.7)
	Transgender	7 (0.1)
	Gender Queer	11 (0.1)
	not completed	2,794 (27.4)
Race (%)	White	5,640 (55.3)
	Black or African-American	536 (5.3)
	Asian	458 (4.5)
	Native Hawaiian or other Pacific Islander	25 (0.2)
	American Indian or Alaska Native	24 (0.2)
	Prefer not to say	1 (0.0)
	Other	625 (6.1)
	Not Completed	2,896 (28.4)
Age Group (%)	18-24	548 (5.4)
	25-34	3,687 (36.1)
	35-44	3,242 (31.8)
	45-54	1,583 (15.5)
	55-64	766 (7.5)
	65-74	286 (2.8)
	75-84	78 (0.8)
	85+	15 (0.1)
Total Duration with Parsley (%)	0 to 1 year	7,390 (72.4)
	1 to 2 years	1,934 (19.0)
	3 or more years	880 (8.6)

**Table 2:** Common conditions and diseases.

Disease Group	*n* (%)	Common ICD-10 Conditions Per Group	Group Inclusion Criteria
Digestive Diseases	5,586 (54.7%)	K58.2 (Mixed irritable bowel syndrome), K90.0 (Celiac disease)	Ever diagnosed with diseases of the digestive system ICD-10 codes (K%)
Skin Diseases	3,909 (38.3)	L65.9 (Nonscarring hair loss), L40.52 (Psoriatic arthritis)	Ever diagnosed with diseases of the skin and subcutaneous tissue ICD-10 codes (L% or between R20-R24)
Cardiometabolic Diseases	4,026 (39.4%)	E78.5 (Hyperlipidemia), I10 [Essential (primary) hypertension]	Ever diagnosed with diseases of the circulatory system and metabolism ICD-10 codes (I% or between E70 and E89)
Hormone and Fertility Diseases	3,750 (36.7%)	E11.9 (Type 2 diabetes mellitus), E10.9 (Type 1 diabetes mellitus)	Ever diagnosed with diseases of the circulatory system (I%) and metabolism (E%) ICD-10 codes
Autoimmune Diseases	2,040 (19.9%)	M06.9 (Rheumatoid arthritis), M10.9 (Gout)	Ever diagnosed with autoimmunity (M%) and related disorders ICD-10 codes
Mental Disorders	4,506 (44.1%)	F41.9 (Anxiety Disorder), F32.9 (Major Depressive Disorder)	Ever diagnosed with mental, behavioral, and neurodevelopmental disorders ICD-10 codes (F%)
Thyroid Disorders	1,912 (18.7)	E02 (Subclinical iodine-deficiency hypothyroidism), E05.00 (Grave's disease)	Ever diagnosed with thyroid disorders ICD-10codes (E00-E08 or T31.8)

**Table 3:** Top 50 ICD-10-CM codes.

ICD-10 CM	Frequency	Code Description
E55.9	14,552	Vitamin D deficiency, unspecified
Z00.01	12,569	Encounter for general adult medical exam w abnormal findings
R53.83	9,320	Other fatigue
R14.0	9,040	Abdominal distension (gaseous)
F41.9	7,100	Anxiety disorder, unspecified
R63.5	4,652	Abnormal weight gain
E72.19	4,130	Other disorders of sulfur-bearing amino-acid metabolism
K59.00	4,057	Constipation, unspecified
K58.0	3,899	Irritable bowel syndrome with diarrhea
R53.82	3,722	Chronic fatigue, unspecified
G47.00	3,458	Insomnia, unspecified
R14.3	3,445	Flatulence
N94.3	2,800	Premenstrual tension syndrome
K21.9	2,592	Gastro-esophageal reflux disease without esophagitis
E03.9	2,499	Hypothyroidism, unspecified
R51	2,333	Headache
E23.3	2,278	Hypothalamic dysfunction, not elsewhere classified
N92.6	2,203	Irregular menstruation, unspecified
E78.5	2,141	Hyperlipidemia, unspecified
E06.3	2,124	Autoimmune thyroiditis
L70.9	1,982	Acne, unspecified
E53.8	1,933	Deficiency of other specified B group vitamins
E78.2	1,900	Mixed hyperlipidemia
K58.9	1,897	Irritable bowel syndrome without diarrhea
R79.82	1,853	Elevated C-reactive protein (CRP)
R19.7	1,766	Diarrhea, unspecified
N94.6	1,736	Dysmenorrhea, unspecified
K58.2	1,728	Mixed irritable bowel syndrome
R68.82	1,672	Decreased libido
E61.1	1,672	Iron deficiency
L20.9	1,652	Atopic dermatitis, unspecified
M25.50	1,609	Pain in unspecified joint
M54.5	1,537	Low back pain
F34.1	1,458	Dysthymic disorder
E53.9	1,422	Vitamin B deficiency, unspecified
L65.9	1,398	Nonscarring hair loss, unspecified
E28.2	1,360	Polycystic ovarian syndrome
J30.9	1,301	Allergic rhinitis, unspecified
L70.0	1,297	Acne vulgaris
Z00.00	1,263	Encntr for general adult medical exam w/o abnormal findings
F41.1	1,233	Generalized anxiety disorder
Z77.120	1,188	Contact with and (suspected) exposure to mold (toxic)
J30.2	1,134	Other seasonal allergic rhinitis
A04.9	1,128	Bacterial intestinal infection, unspecified
Z91.018	1,102	Allergy to other foods
F90.9	1,072	Attention-deficit hyperactivity disorder, unspecified type
K90.41	1,049	Non-celiac Gluten Sensitivity
G31.84	1,045	Mild cognitive impairment, so stated
K90.89	1,039	Other intestinal malabsorption
L70.8	1,033	Other acne

### Efficiency

**Figure 3:**
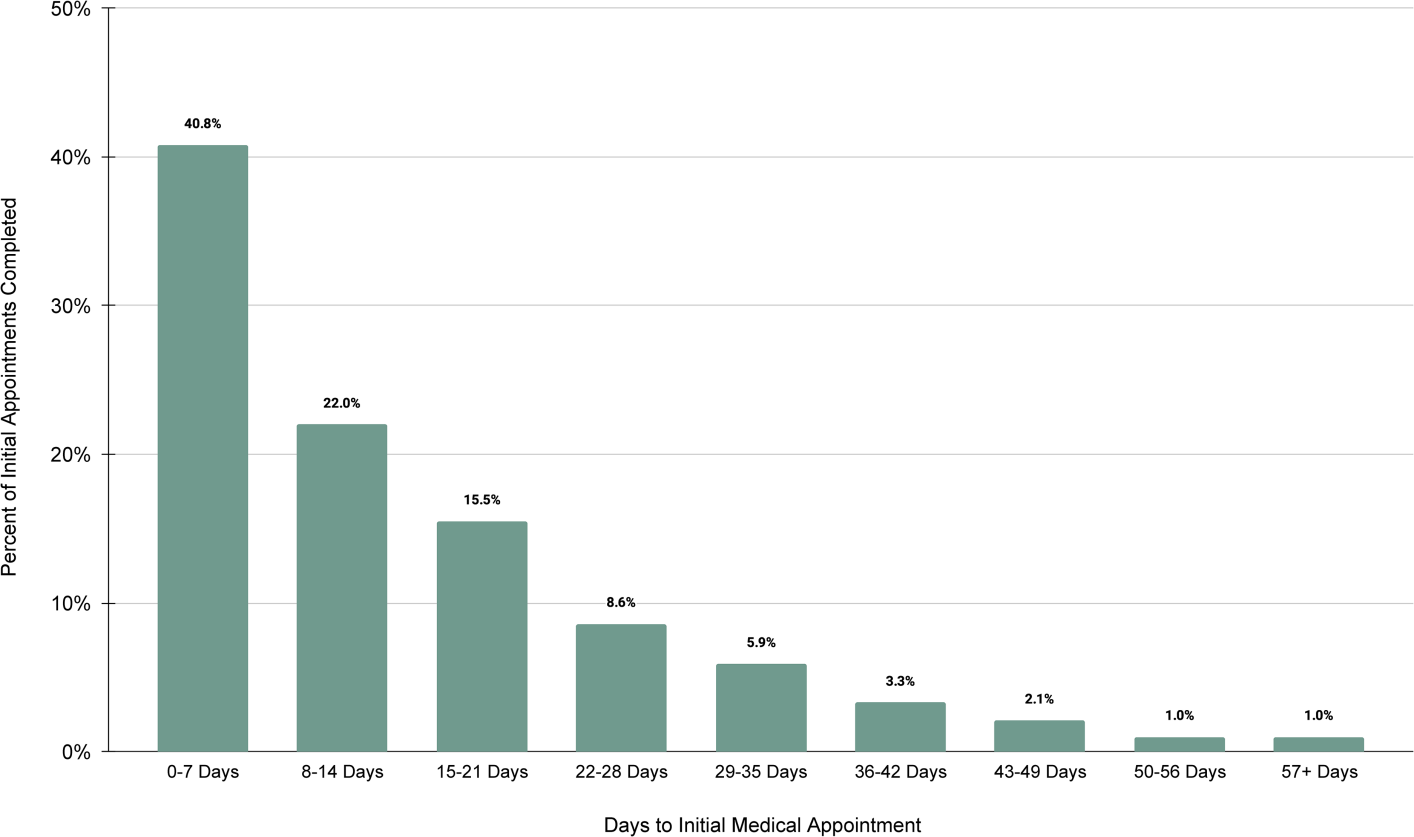
Days to initial medical appointment.

Efficiency After enrolling in a care plan, 62.8% of patients had their initial appointment with a clinician within two weeks of scheduling. The vast majority of patients (86.9%) were able to complete their first visit within 30 days (Figure 3).

### Feasibility

The average number of visits with a clinician and health coach varied by care plan. In the first year of membership, patients completed an average of 4.8 visits. This included clinician and health coach visits.

Patients also had high rates of initial PSI completion (97.3%) in preparation for the first visit with a care provider. The PSI on average takes less than five minutes to complete (26), and was required before the initial clinician visit, so this number was expected to be high. Completion rates were lower for subsequent encounters, but the PSI completion rate averaged 80.4% per month over the course of the study period.

### Acceptability

**Figure 4:**
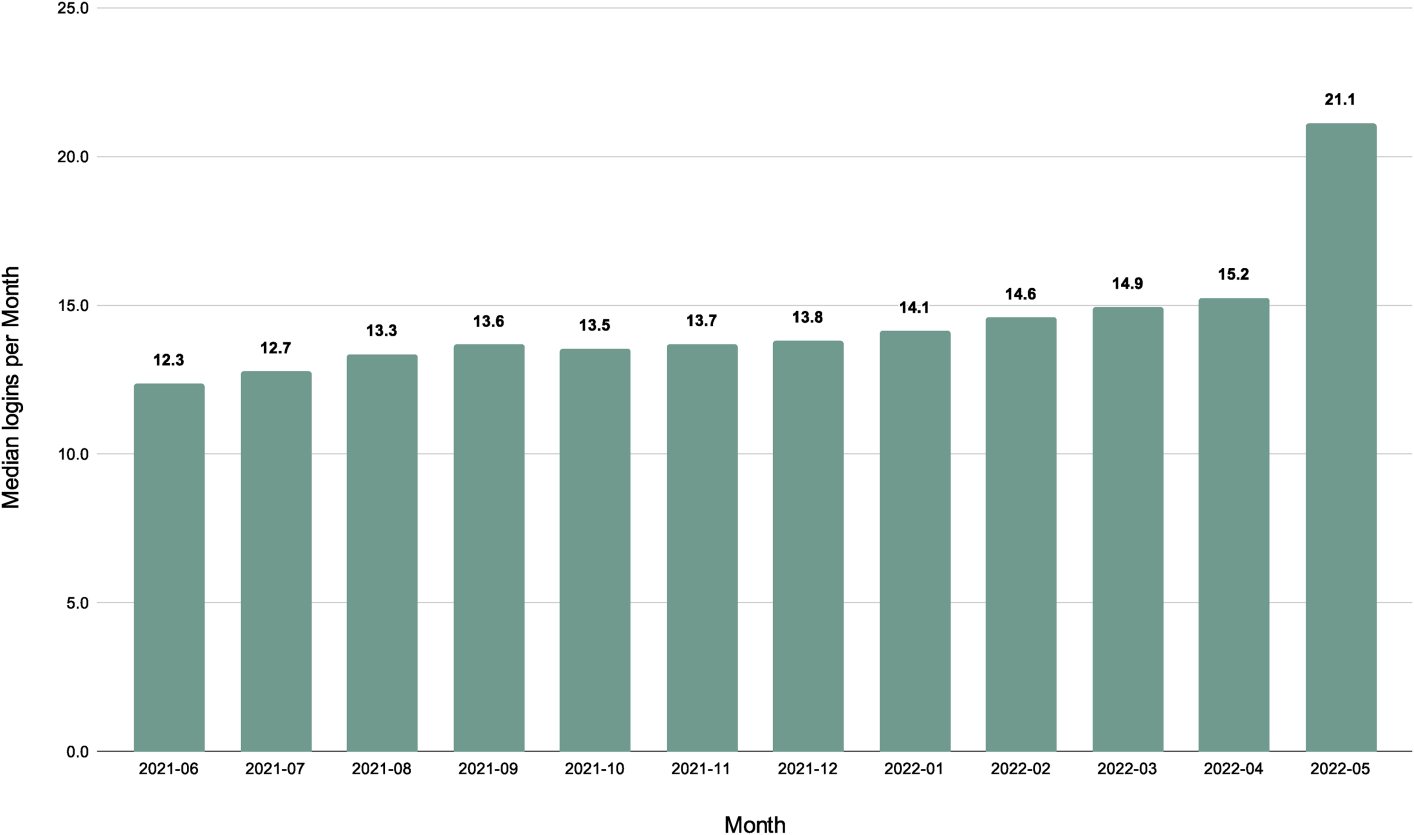
Median logins per month.

Patients accessed the MyParsley portal an average of approximately 14 times per month to schedule visits, review visit notes, and view test results. Between June 2021 and June 2022, the average number of logins range from a monthly average of 12.45 to 16.53 due to usability improvements made to the website (Figure 4).

**Figure 5:**
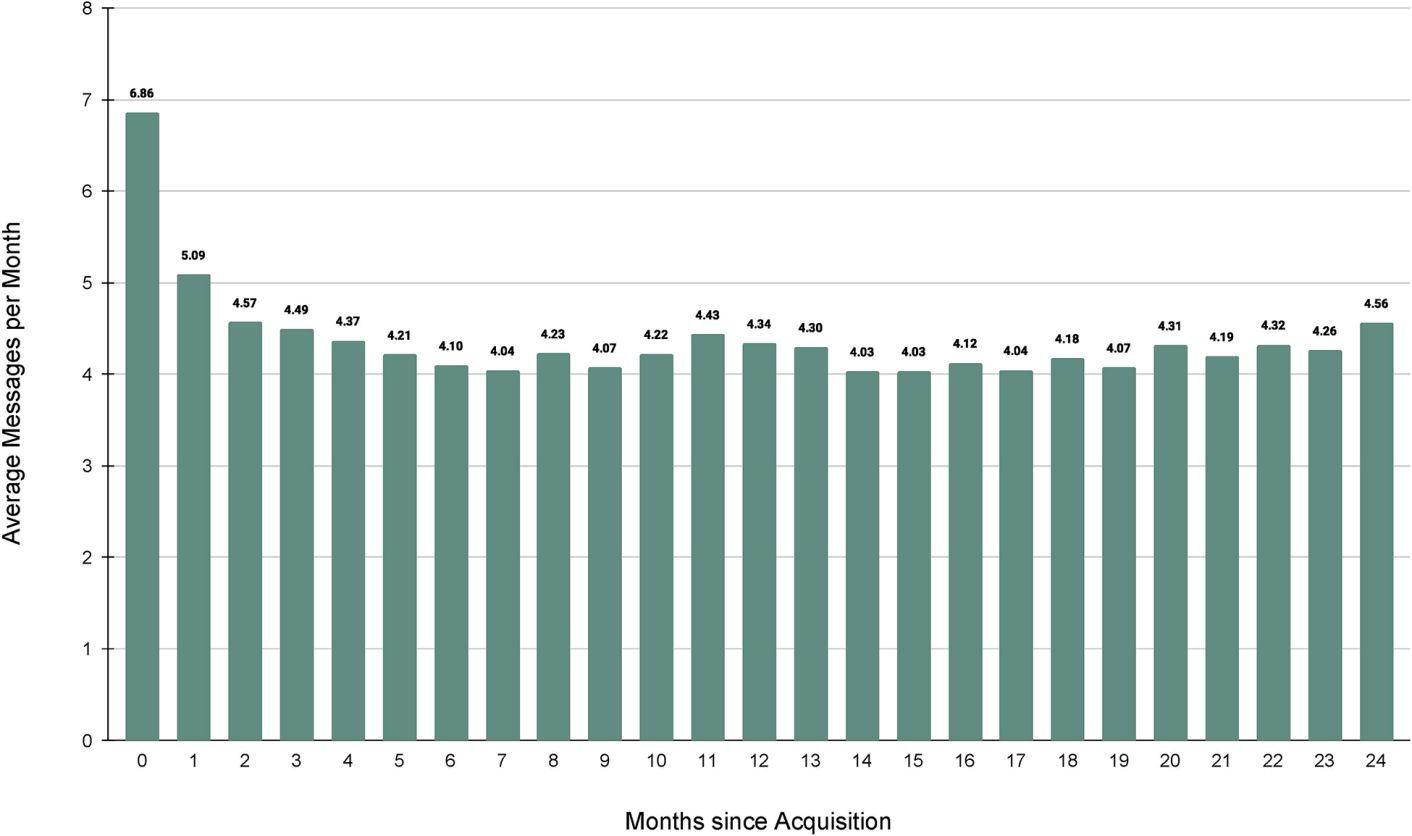
Average messages per month.

Patients also regularly utilized MyParsley to send messages to their care team. The average number of monthly messages sent ranged from 4 to 7, with overall messaging remained relatively consistent during a patient's membership (Figure 5).

### Satisfaction

**Figure 6:**
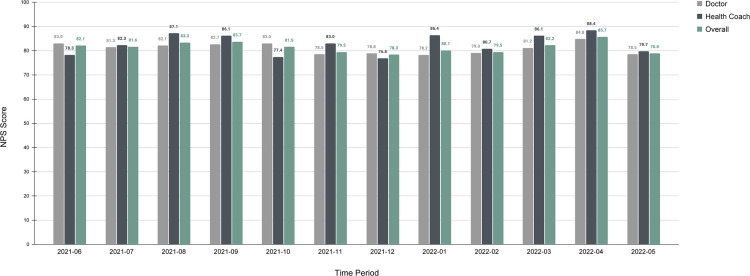
NPS scores for doctors, health coaches, and combined global scores.

Both in-person and telehealth patients completed net promoter score (NPS) on a regular basis. All patients were offered an NPS survey to fill out after every clinician and health coach visit. Across all clinical encounters, the NPS was completed 38.1% of the time. This represents 6,008 NPS forms filled out for 15,245 clinical encounters. Over the period of June 1, 2021 to June 1, 2022, the average NPS score for doctors was 80.92, for health coaches was 82.70 with a global average NPS score of 81.35 (Figure 6) based on a sample size of 6,008 responses. While the difference between the doctor and health coach average is not substantial, it is notable that the addition of the health coach visits increases the average NPS score. Also of note, compared to the Health Coach NPS scores, the Doctor NPS scores represent a larger number of yearly responses (N 4549 vs. N 1459).. A subset of members (*N* = 200) completed customer satisfaction (CSAT) surveys between January 2022 and April 2022. The CSAT survey was offered every 2 weeks to all members who had either a clinician or health coach visit in the past 55 days. No incentive was offered for NPS or CSAT completion. Because the subjects were deidentified, demographic data is not available. Of these respondents 100% agreed with the statement “My Parsley team cares about me”, with the vast majority agreeing with the following statements: “My care team is guiding me towards my health goals” (94%), “My care team is knowledgeable” (96%), “I know what steps I need to take to improve my health” (91%), “I feel better” (93%), “Setting up my MyParsley online member account was simple” (95%), “I can find information on my MyParsley online account with ease” (96%), and “Scheduling my visits with my Clinician and my Health Coach is easy” (87%).

### Effectiveness

**Figure 7:**
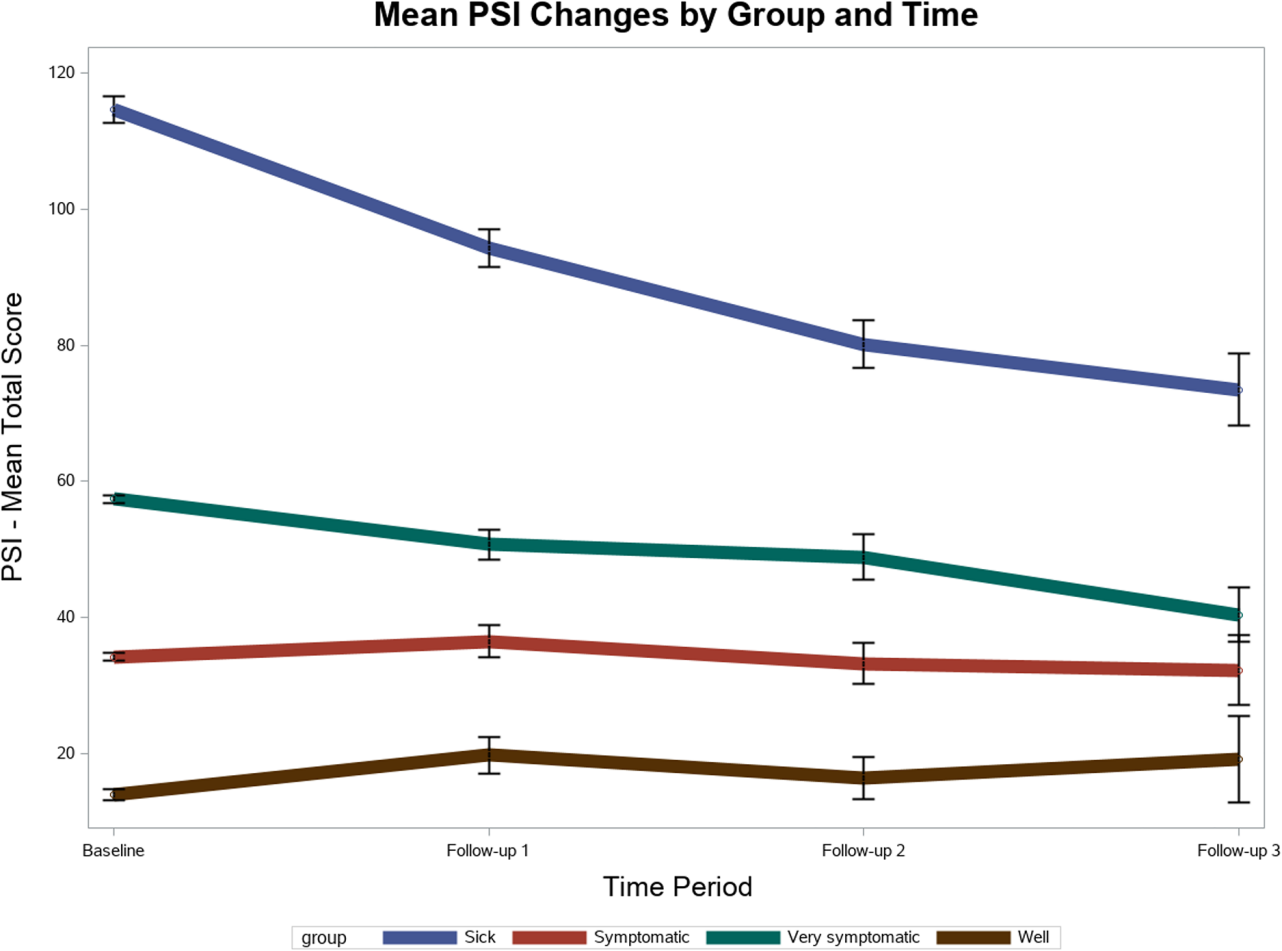
PSI improvement Over Time.

A total of 2,897 unique participants had completed an initial baseline PSI on or after June 1st 2021. The initial baseline PSI total scores led to the following group distributions for the efficacy analysis: 49.91% (2,996) of participants as sick, 25.68% (744) as very symptomatic, 14.26% (413) as symptomatic, and 10.15% (294) as well. In total 5,984 PSI's were included between the initial baseline period and three follow-up periods that were separated each by an interval period of approximately three months. As described in Table 4 and visualized in Figure 7, PSI mean scores significantly improved between each time point for the sick and very symptomatic groups. The largest within-group improvements occurred between baseline and follow-up three for the sick group, with a statistically significant Least Squares (LS) mean decrease of −39.27 (SE = 1.58, CI, 36.16 to 42.38, *p* < . 0001), and the very symptomatic group with a LS mean decrease of −17.83 (SE = 2.13, CI, 13.66, 22.01, *p* < 0001). The only observed significant improvement in the well group was between follow up 1 and follow up 3 with a LS mean estimate reduction of −2.69 (SE = 4.12, CI, −10.77 to 5.38, *p* = .05). No significant improvements were detected within the symptomatic group across time.

**Table 4:** Unadjusted means and standard deviations and model adjusted means and standard errors for PSI group by time.

	Well			Symptomatic			Very Symptomatic			Sick		
	**Unadjusted**											
PSI	Mean	SD	95% CI	Mean	SD	95% CI	Mean	SD	95% CI	Mean	SD	95% CI
Baseline	14.00	0.43	13.15, 14.85	34.22	0.27	33.68, 34.75	57.38	0.30	56.79, 57.96	114.67	0.99	112.72, 116.61
Follow-up 1	19.77	1.39	17.02, 22.52	36.53	1.22	34.12, 38.94	50.77	1.12	48.57, 52.96	94.27	1.45	91.42, 97.10
Follow-up 2	16.43	1.54	13.37, 19.48	33.22	1.52	30.20, 36.22	48.89	1.67	45.59, 52.17	80.16	1.81	76.61, 83.70
Follow-up 3	19.21	3.14	12.82, 25.60	32.30	2.53	27.24, 37.35	40.44	2.01	36.44, 44.43	73.48	2.70	68.15, 78.79
	**Adjusted**											
PSI	Mean	SE	95% CI	Mean	SE	95% CI	Mean	SE	95% CI	Mean	SE	95% CI
Baseline	14.00	1.76	10.55, 17.45	34.22	1.48	31.30, 37.12	57.38	1.11	55.21, 59.54	114.67	0.95	113.12, 116.23
Follow-up 1	19.38	2.14	15.18, 23.58	36.24	1.77	32.76, 39.71	50.60	1.31	48.02, 53.17	94.65	1.16	92.78, 96.50
Follow-up 2	16.50	2.74	11.12, 21.87	32.52	2.26	28.09, 36.94	48.24	1.62	45.06, 51.41	81.89	1.63	79.61, 84.17
Follow-up 3	19.20	3.99	11.37, 27.02	31.46	2.89	25.78, 37.13	39.54	2.19	35.24, 43.83	75.40	1.48	72.20, 78.58

## Discussion

Data collected on patient engagement, satisfaction, and preliminary self-reported outcomes highlight the feasibility, acceptability, and potential effectiveness of the Parsley Health holistic program for chronic disease management.

### Efficiency

People with chronic disease often experience long wait times to receive a diagnosis (50) or to see a specialist once diagnosed (51). Patients in this study were able to schedule an appointment quickly and efficiently, with the majority scheduling an appointment within a month of signing up for the program. Quickly scheduling their first visit indicates the online platform patients use to schedule appointments (whether in person or virtual) is easy to use, making the process more efficient for patients, and that patients were readily engaged with their membership.

The initial clinician visit was also a more involved process and experience for patients. This was largely because the intake forms were more in-depth than those used in typical medical encounters. In a holistic medical evaluation, the clinician reviews all the intake forms and takes a detailed medical, psychosocial and environmental history starting at birth, with an eye to identifying antecedents, triggers, and mediators of disease states. The clinician then tells the story back to the patient, which is often a very moving and thought-provoking process. The significant time participants devoted, coupled with an efficient process for seeing a clinician in a timely fashion, reinforced patient satisfaction and leveraged a participant's momentum and motivation to continue in the program and actively engage with their clinicians in subsequent visits.

### Feasibility

Patients did not use all available visits in their care plan. For patients who found that they didn't need all 10 visits over the course of the year, they could sign up for a renewal plan with fewer visits. Notably, the average number of visits to a primary care provider among U.S. adults is less than three (52), suggesting that Parsley Health patients utilized more healthcare services than the national average.

There were likely many factors impacting utilization. For one, patients were able to engage with a member experience employee, who helped ensure patients in their first year chose a care team that was the right fit for their personal and medical goals. Because patients were carefully matched, they showed up regularly for their visits. Depending on the complexity of their disease, some patients needed less care. For those with less complex diseases and those who see significant improvement over the course of their first year, they could opt for renewal plans with less frequent follow-up.

Completing the PSI ensured that patients and providers had a shared, data-informed touchpoint to enhance each health visit and track symptoms over time. PSI completion rates have always been highest at the first visit when it was required to see the clinician (26). While the completion rates decreased after the first visit, the overall completion rate remained high across all visits. A prior validation study showed that patients completed the PSI efficiently in under 10 min (26). High PSI completion demonstrated positive participant response to the intervention and a willingness to engage.

### Acceptability

In addition to completing the PSI and showing up to visits, participants utilized other tools to remain engaged in their care. The ability to message care teams when questions arose and monitor results of tests or forms like the PSI also gave patients access to their own data to track trends over time, making it easier to manage their own health.

Moreover, patients found the digital tools and interfaces used in the care program to be user-friendly. On the MyParsley portal, participants reported that setting up an account, accessing key information about their membership or their visits, and scheduling appointments were all done with relative ease. The availability of member experience and care managers improved the usability of these tools.

The use of digital tools at the center of the care program also illustrated the adaptability of the program to meet patient needs and preferences. All intake forms and health records were stored and completed online, and all visits were virtual for the majority of the COVID-19 pandemic. As the pandemic has evolved, some patients have chosen in-person visits while many have elected to continue virtual care, effectively making the model a hybrid healthcare option for patients.

### Satisfaction

**Figure 8:**
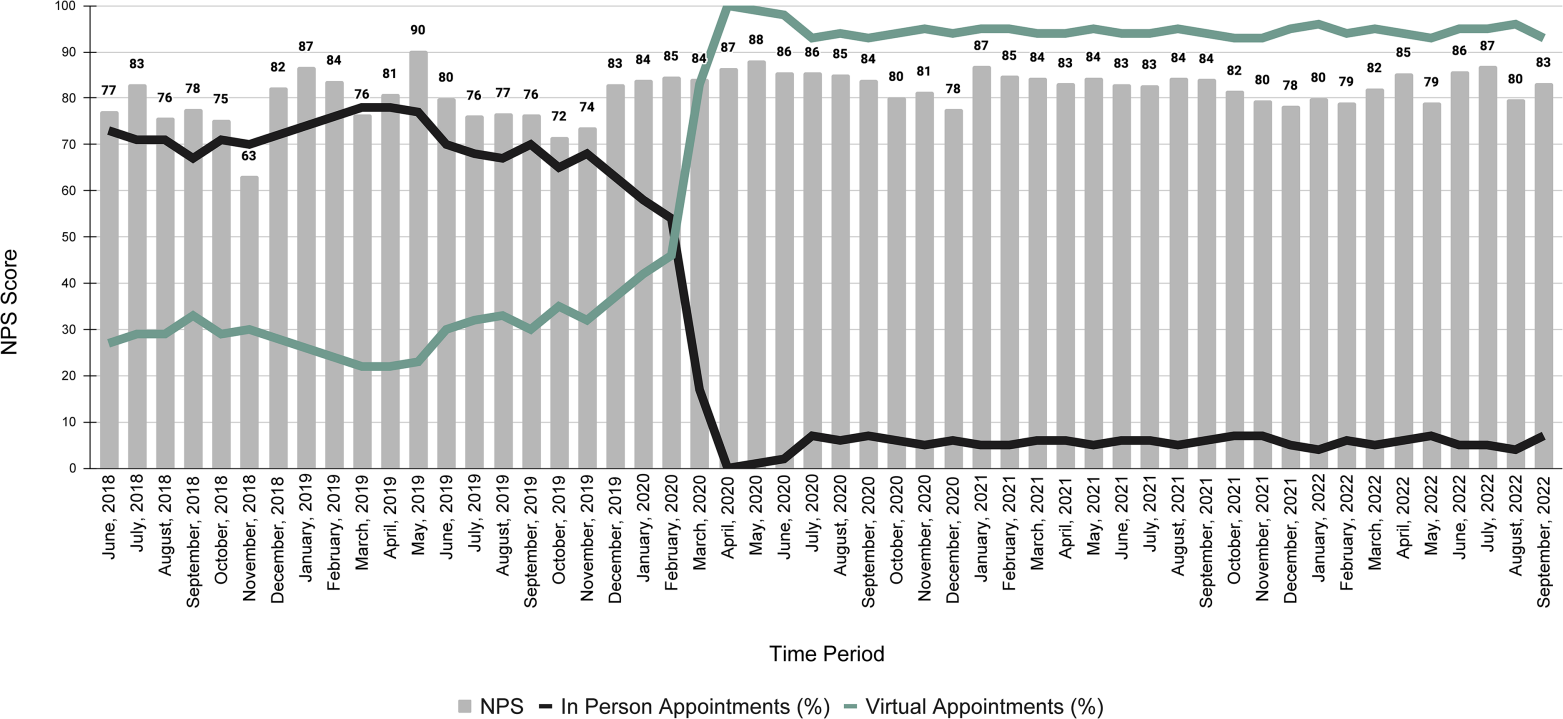
Virtual versus in-person visit usage and global NPS scores.

Patient satisfaction is important to the success of a care practice (53). It is equally important in driving clinical effectiveness (54). The notion of satisfaction is particularly powerful given the role satisfaction plays in the successful implementation of chronic care interventions (55). Global average NPS scores were consistently excellent, and well above national averages for health care and telehealth. The U.S. healthcare industry standard is about 58, and the average NPS score for telehealth with video is about 70 (56). NPS scores were de-identified after visits, so we cannot say which NPS scores correspond to in-person visits vs. virtual visits. However, Figure 8 shows that in March 2020 (pre-study period data), at the onset of the COVID-19 pandemic, the ratio of in person to virtual visits flipped abruptly. Prior to the pandemic, approximately 60%–70% of visits were in-person and 20%–30% were virtual. After the onset of the pandemic, in-person visits fell to 0%–7% and virtual visits accounted for 90%–100% of all visits. Throughout the pandemic when most patients used virtual services, NPS scores stayed steady, ranging from 77.6–88.2. Prior to the pandemic NPS scores ranged from 63.1–90.32. In general, NPS scores improved and were more stable after the pandemic. This was in part due to the increase in sample size.

The CSAT survey results demonstrated Parsley Health patients were highly satisfied with all aspects of the program, from ease of use, to feeling that their care team was knowledgeable and guiding them on a shared journey toward meeting their health goals. Ultimately, the program's set up helped build patient-provider trust, which is associated with improved health outcomes (57). Overall, patients felt that their healthcare team was responsive to their needs and that the care they received gave them the resources to actively manage their own care.

### Effectiveness

Participants also reported significantly improved self-reported symptom severity over the course of a year in the care program. This was a positive for patients with chronic conditions, as high symptom burden can reduce quality of life (58). With more frequent visits, regular health data monitoring, and ongoing communication, we believe the Parsley Health program created a positive feedback loop that led to self-reported improvements in a patient's overall health, providing preliminary evidence of the program's effectiveness as a chronic condition care model.

### Limitations

The Parsley Health patients captured within this study were predominantly white and female, and not reflective of the sociodemographic diversity that exists in the United States, limiting the generalizability of our results. However, it is representative of the Parsley Health patient population. Second, the included implementation and feasibility measures were originally adopted for the purposes of monitoring key business performance indicators for the Parsley Health program, and not with the *a priori* intent of conducting a research study. Third, because telehealth is still an emerging health care standard, there is a lack of data about other telehealth programs to offer comparison to Parsley Health, which would have improved the analysis of the program's feasibility. We hope this paper inspires more research to provide data for comparison. Lastly, this study contains only one data point (PSI score reduction) on symptom improvement as reported by patients, and does not include any additional objective disease measurements such as lab or imaging studies that could be used to corroborate patient self-reports. Future studies will address health outcomes specifically in more depth.

### Conclusion

In our current healthcare system, patients with chronic conditions receive less care than they need, spend less time with a physician to address their questions, and have poor health outcomes. The onset of chronic disease is often viewed as the beginning of an inevitable decline rather than an opportunity to reverse course. As a result, there is a demand for new healthcare options among people with chronic conditions, including a demand for holistic, preventive chronic disease care. Parsley Health's approach to delivering a blend of holistic and usual approaches to care through the unique affordances of digital tools allowed people with chronic conditions to access care. While we know that telehealth tools can be used to deliver effective chronic care interventions, there has been little research looking at how to design and deliver these interventions on a large scale and still be effective.

Amid challenges with studying transformations in the delivery of health care (specifically, appropriate research methodologies) (59), the blending of various outcome metrics helps highlight that Parsley Health has potential as a transformative, large-scale telehealth program for delivering chronic disease care. Findings from this study suggest that the program was successfully implemented, as evidenced by participant engagement with the program and overall satisfaction with the care they received as indicated by NPS scores, which were substantially higher than the national average for telehealth. Patient satisfaction and engagement likely contributed to consistently improved symptom severity, as indicated by improved PSI scores over time, though more research is needed to understand treatment and clinical-related outcomes of the program. Ultimately, the Parsley Health patients represented in this study felt cared for, heard by their providers, and felt much better after a year of receiving care *t*.

## Data Availability

The raw data supporting the conclusions of this article will be made available by the authors, without undue reservation.
